# Dynamic Analysis and Reservoir Computing Application of a Nonlinear Microring Resonator

**DOI:** 10.1021/acsphotonics.5c00681

**Published:** 2025-08-25

**Authors:** Stefano Gretter, Mattia Mancinelli, Lorenzo Pavesi

**Affiliations:** Nanoscience Laboratory, Department of Physics, University of Trento, Via Sommarive, 14, 38123 Povo, Trento, Italy

**Keywords:** nonlinear microring resonator, local stability analysis, reservoir computing, Jacobian eigenvalues, dynamical systems

## Abstract

A nonlinear microring resonator is governed by a set of coupled differential equations that model the dynamics of the optical field, temperature, and free carrier concentration within the resonator. These equations capture the mechanisms responsible for self-pulsing and memory effects, which are key in neuromorphic applications of microring resonators. One example is their use as nonlinear nodes in reservoir computing (RC). The dynamical state of a microring resonator is influenced by its control parameters: the input optical power and frequency. While previous studies have relied heavily on computationally intensive simulations to determine the resonator’s self-pulsing state or identify optimal control parameters for efficient optical computation, we propose a linearization and stability analysis to identify regions in the control parameter space associated with different dynamical behaviors. Using an adiabatic approximation of the cavity field of the mode, we calculated the Jacobian eigenvalues of the linearized system, which serve as reliable indicators of RC performance for specific input characteristics.

## Graphical abstract

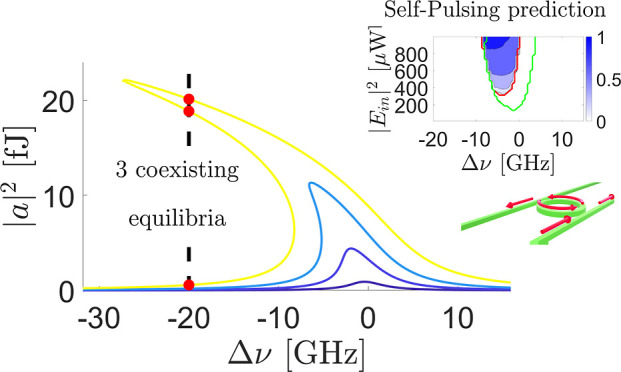

## 1 Introduction

Microring resonators (MRRs) are highly valued in photonic neural networks due to their compact size, passive nature, and easily accessible nonlinear effects. These characteristics make them versatile components for optical computing.^1^ Indeed, in neural networks they are capable of solving logical and analog tasks,^2–5^ as well as they can be used in time series processing^6^ or as spiking neurons.^7^ The high compactness of MRRs enables large arrays^8^ with different degrees of recurrence or memory.^9^ However, the increased complexity of these arrays poses significant computational challenges, particularly in identifying the optimal control parameters for a given input signal. To address this challenge, we propose to describe the MRR dynamics with a linearization and stability analysis—a technique commonly applied to complex systems.^10^

In this paper, we concentrate on single MRRs and validate the proposed method to study their fading memory properties.^4^ We discuss the MRR dynamical time scales as a function of the power and frequency of the input signal, i.e., the control parameters. Additionally, we study the stability in the dynamics of the optical field of the MRR optical mode. In fact, when sufficient continuous-wave (CW) power is injected into a MRR, its transmission exhibits a periodic oscillatory dynamic known as self-pulsing.^11,12^ This phenomenon arises from the interplay of competing nonlinear effects: free carrier dispersion and thermo-optic effects. By applying a linearization and stability analysis, we identified the self-pulsing region in the control parameter space.

This paper is organized as follows: first, we introduce the nonlinear MRR and the linearization and stability analysis. Then, we outline the procedure for linearization and stability analysis of the equilibrium point at the low energy state of a MRR, comparing the analytical results with numerical solutions obtained using the Runge–Kutta method. Moreover, we examine the other equilibrium points and their influence on the MRR’s dynamics. Finally, we investigate the MRR’s functionality as a neural network, offering insights into the selection of control parameters that optimize task performance through eigenvalue maps.

## 2 Results and Discussion

### 2.1 The Dynamics of a Microring Resonator

The sketch in Figure 1a shows the general scheme of a MRR in the add-drop configuration.^13–15^

**Figure 1:**
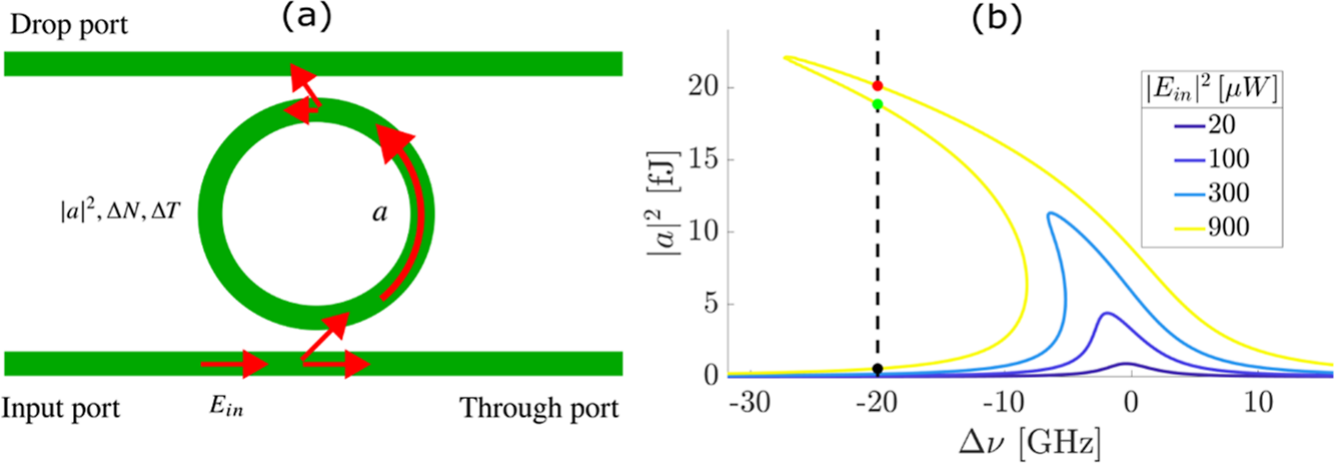
(a) A sketch of a MRR in the add-drop configuration. The red arrows show the path of the optical field inside the bus waveguides and the MRR. The different symbols refer to *E*_in_ input field, a optical mode field in the MRR, Δ*T* temperature increase, Δ*N* free carrier concentration increase, |*a*|^2^ optical energy stored in the MRR. (b) Nonlinear twisted Lorentzian profile of the equilibrium cavity energy |*a*|^2^ as a function of the detuning Δν from the MRR cold resonance for different input powers (*P*_in_ = |*E*_in_|^2^). The black, green, and red markers refer to the lowest, middle, and highest energy equilibrium points. They correspond to −20 GHz and 900 μW control parameters.

The physics and the modeling of MRRs are described in refs 1,13 . Here, suffices to say that the input signal can excite free carriers in the MRR through two-photon absorption. This modifies the free carriers concentration in the MRR by a quantity Δ*N* and, consequently, the real and imaginary parts of the refractive index. Moreover, the temperature of the MRR increases by Δ*T* because of free carrier relaxation and light absorption, which shifts the real part of the refractive index. A variation in the refractive index changes the energy stored in the MRR. To include these power-dependent (nonlinear) effects in the modeling of the dynamics of a nonlinear MRR, it is customary to consider a system of 4 real (2 real and 1 complex) first-order coupled nonlinear differential equations^16^

1a $$\frac{da}{dt}=\left[i\left(\omega_{r}+\delta \omega_{nl}-\omega \right)-\frac{\gamma_{loss}}{2}\right]a+i\sqrt{\gamma_{coup}}E_{in}$$

1b $$\frac{d\Delta N}{dt}=\frac{\Gamma_{FCA}\beta_{Si}}{2\hbar \omega V_{FCA}^{2}}\frac{c^{2}}{n_{g}^{2}}{\left|a\right|}^{4}-\frac{\Delta N}{\tau_{fc}}$$

1c $$\frac{d\Delta T}{dt}=\frac{\Gamma_{th}\gamma_{abs}}{\rho_{Si}c_{p,Si}V_{th}}|a{|}^{2}-\frac{\Delta T}{\tau_{th}}$$

where

2a $$\gamma_{loss}=2\gamma_{coup}+\gamma_{rad}+\gamma_{abs}$$

2b $$\gamma_{abs}=\gamma_{abs,lin}+\frac{\Gamma_{TPA}\beta_{Si}}{V_{TPA}}\frac{c^{2}}{n_{g}^{2}}{\left|a\right|}^{2}+\Gamma_{FCA}\sigma_{Si}\frac{c}{n_{g}}\Delta N$$

2c $$\delta \omega_{nl}=-\frac{\omega_{r}}{n_{g}}\left(\frac{dn_{Si}}{dT}\Delta T+\frac{dn_{Si}}{dN}\Delta N\right)$$

In eq 1a, *a* is the slow envelope of the complex cavity mode field amplitude. The other quantities are introduced and tabulated in Appendix A.4. Note that, if the MRR geometry is an all-pass geometry, the only needed change is to remove the factor 2 in the definition of γ_loss_ in eq 2a. For a detailed discussion of this equation, the reader is referred to the literature.^1,13,17^

In the linear regime, i.e., when the input optical power *P*_in_ = |*E*_in_|^2^ is low, the solution of eq 1a is a Lorentzian line shape for |*a*|^2^. As we enter the nonlinear regime, |*a*|^2^ departs from the Lorentzian line shape. Increasing *P*_in_, one can numerically solve the system of eqs 1a–1c and get the nonlinear evolution of |*a*|^2^. Figure 1b shows |*a*|^2^ at equilibrium for various *P*_in_. Appendix A.2 clarifies the method we used to compute |*a*|^2^ in stationary conditions. As already reported in ref 13, the resonance line shape is close to the linear Lorentzian profile for low-injected powers. While it becomes twisted for high *P*_in_, namely when nonlinear effects become relevant, and leans to the negative detuning frequencies (Δν = (ω_r_ – ω)/2π where ω_r_ is the MRR cold resonance angular frequency).

Let us note that for the MRR under consideration, which has a radius of about 7 μm and an external coupling coefficient γ_coup_ = 9.8 MHz, the optical mode *a* reaches a stationary state in a time scale (∼10 ps). This time is much shorter than the characteristic times of the nonlinear dynamical effects (the free carriers and temperature time scales are ∼10 ns and ∼100 ns, respectively). This allows simplifying eq 1a by setting $\frac{da}{dt}=0$ .^18^ Note that, even under such an approximation, *a* implicitly depends on time through its dependence on Δ*N*(*t*) and Δ*T*(*t*). By setting the derivative to zero in eq 1a, we can evaluate |*a*|^2^ as a function of Δ*N* and Δ*T* (see Appendix A.1), reducing the system of 4 equations to a system of two equations (eq ([Disp-formula eq1b]) and eq ([Disp-formula eq1c])). By using the method reported in Appendix A.2, we evaluate the equilibrium values Δ*N*_eq_, Δ*T*_eq_ as a function of the control parameters (*P*_in_ and Δν). The system has at least one, and at most three equilibrium points in the (Δ*N*, Δ*T*) space (see Appendix A.2). Then, we linearize eqs 1b and 1c at one equilibrium point^10^

3 $$\begin{array}{ll} \frac{d\Delta N}{dt} & =F_{N}\left(\Delta N,\Delta T\right)\approx F_{N}\left(\Delta N_{eq},\Delta T_{eq}\right)+\frac{\partial F_{N}}{\partial \Delta N}\left(\Delta N-\Delta N_{eq}\right)+\frac{\partial F_{N}}{\partial \Delta T}\left(\Delta T-\Delta T_{eq}\right)\\ \frac{d\Delta T}{dt} & =F_{T}\left(\Delta N,\Delta T\right)\approx F_{T}\left(\Delta N_{eq},\Delta T_{eq}\right)+\frac{\partial F_{T}}{\partial \Delta N}\left(\Delta N-\Delta N_{eq}\right)+\frac{\partial F_{T}}{\partial \Delta T}\left(\Delta T-\Delta T_{eq}\right) \end{array}$$

where we have introduced the two implicit nonlinear functions *F*_T_ and *F*_N_. Note that these functions implicitly depend on |*a*|^2^ via eq 7.

It follows (see ref 10) that as long as the system is close to equilibrium, the linearization is accurate and it holds that

4 $$\left(\begin{array}{l} \Delta N-\Delta N_{eq}\\ \Delta T-\Delta T_{eq} \end{array}\right)=k_{1}\vec{\xi_{1}}e^{\lambda_{1}t}+k_{2}\vec{\xi_{2}}e^{\lambda_{2}t},$$

where ${\mathrm{\xi ⃗}}_{1,2}$ and λ_1,2_ are the two eigenvectors and eigenvalues of the Jacobian of the linearized system (eq 3), while *k*_*i*_, *i* = 1, 2 are real constants fixed by the initial conditions and *t* is time. The solutions of eq 4 describe the dynamics of the system in the proximity of the equilibrium point. As long as the system does not depart from the equilibrium point, the proposed method can be applied. This means that the full computational solution to the system of eq ([Disp-formula eq1a]) is equal to the solutions given by eq 4.

### 2.2 Equilibrium Point at the Lowest Microring Energy

As already mentioned, depending on the control parameters, more than one equilibrium can coexist ((Δ*N*_eq_^*i*^,Δ*T*_eq_^*i*^), *i* = 1, 2, 3). For example Figure 1b shows three equilibrium points for Δν = −20 GHz and *P*_in_ = 900 μW. In the following, we consider the equilibrium corresponding to the lowest MRR stored energy, i.e. the minimum among the three |*a*(Δ*N*_eq_^*i*^,Δ*T*_eq_^*i*^)|^2^. This is the one that is closest to the situation of no field in the MRR for *t* = 0. This initial condition is usually used in experiments. When a different initial condition is used, the system may get closer to another equilibrium point at later times. The dynamics in this case are different and will be discussed in the next subsection.

From eq 4 and the discussion in ref.,^10^ it follows that the stability of the equilibrium point can be determined by the eigenvalues of the system. If at least one eigenvalue has a positive real part (Re­(λ_*i*_) > 0, *i* = 1 or 2), Δ*N* and Δ*T* depart exponentially from the equilibrium, rendering the equilibrium unstable. Conversely, if the real parts of both eigenvalues are negative, the system is stable, meaning that the free carrier concentration and temperature of the MRR converge to their equilibrium values. When the eigenvalues have nonzero imaginary parts, the system exhibits oscillatory dynamics. Consequently, the study of the eigenvalues allows determining the response of the MRR to an input signal. This is summarized in Figure 2 which presents the central findings of this work. Figure 2a shows that three distinct regions are found in the control parameter space: one characterized by oscillatory unstable dynamics (within the red line in Figure 2a), one with oscillatory stable dynamics (exterior to the red and inside the green lines in Figure 2a), and one with nonoscillatory stable dynamics (exterior to the green and red lines Figure 2a). Figure 2b depicts the imaginary part of the first eigenvalue, while Figure 2c,d show the real parts of the first and second eigenvalues as functions of the control parameters. Notably, the imaginary part of the second eigenvalue, Im­(λ_2_), is equal to −Im­(λ_1_), as demonstrated in Appendix A.3.

**Figure 2:**
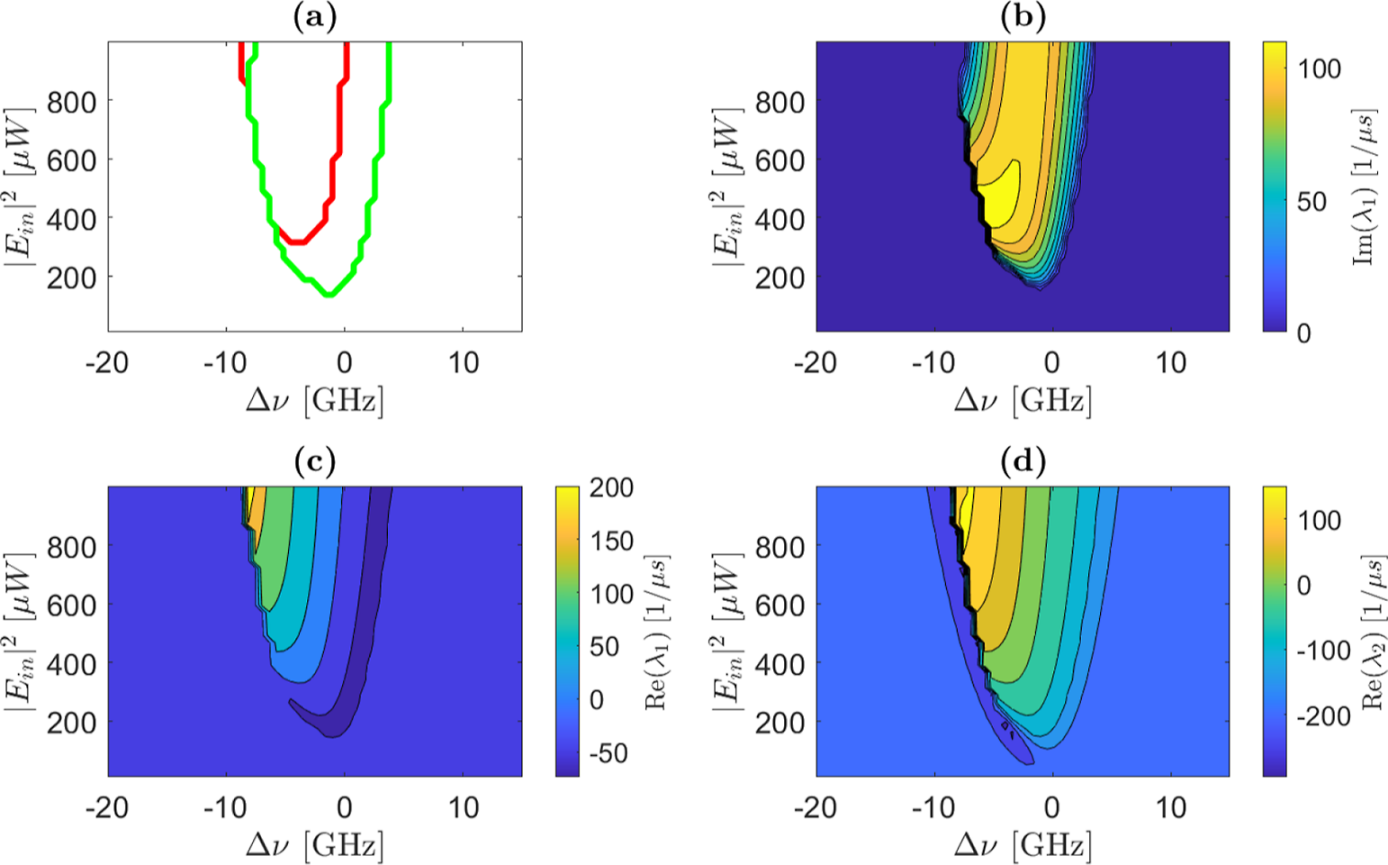
(a) Stability map of the microring as a function of the control parameters (*P*_in_ = |*E*_in_|^2^ input power and Δν detuning with respect to the cold microring resonance). The red and green lines divide the map in three regions. Within the region defined by the red line, at least one eigenvalue has a positive real part. Within the region outside the red and inside the green lines, the imaginary parts of the two eigenvalues are different from zero and the real parts are both negative. The imaginary part is equal to zero in the region exterior to the green line. (b) Map of the imaginary part of the first eigenvalue as a function of the control parameters. (c,d) Maps of the real parts of the first and second eigenvalues, respectively. The color scales are given by the bars on the right of panels (b–d). Note that for the real parts, these span both positive and negative values. The eigenvalues are evaluated at the first equilibrium point.

To validate these results, we solved the nonlinear system in eq 3 by using the Runge–Kutta algorithm and computed the dynamics of the system.^16^ To represent the self-pulsing state, we draw the map of the standard deviation of the field intensity within the ring as a function of the control parameters (Figure 3e). Phase portraits, illustrating trajectories in the Δ*N*–Δ*T* space, are shown in Figure 3d,b,f. Additionally, Figure 3a,c display the temporal evolution of the stored MRR optical energy (|*a*|^2^) for cases (d) and (f), respectively.

**Figure 3:**
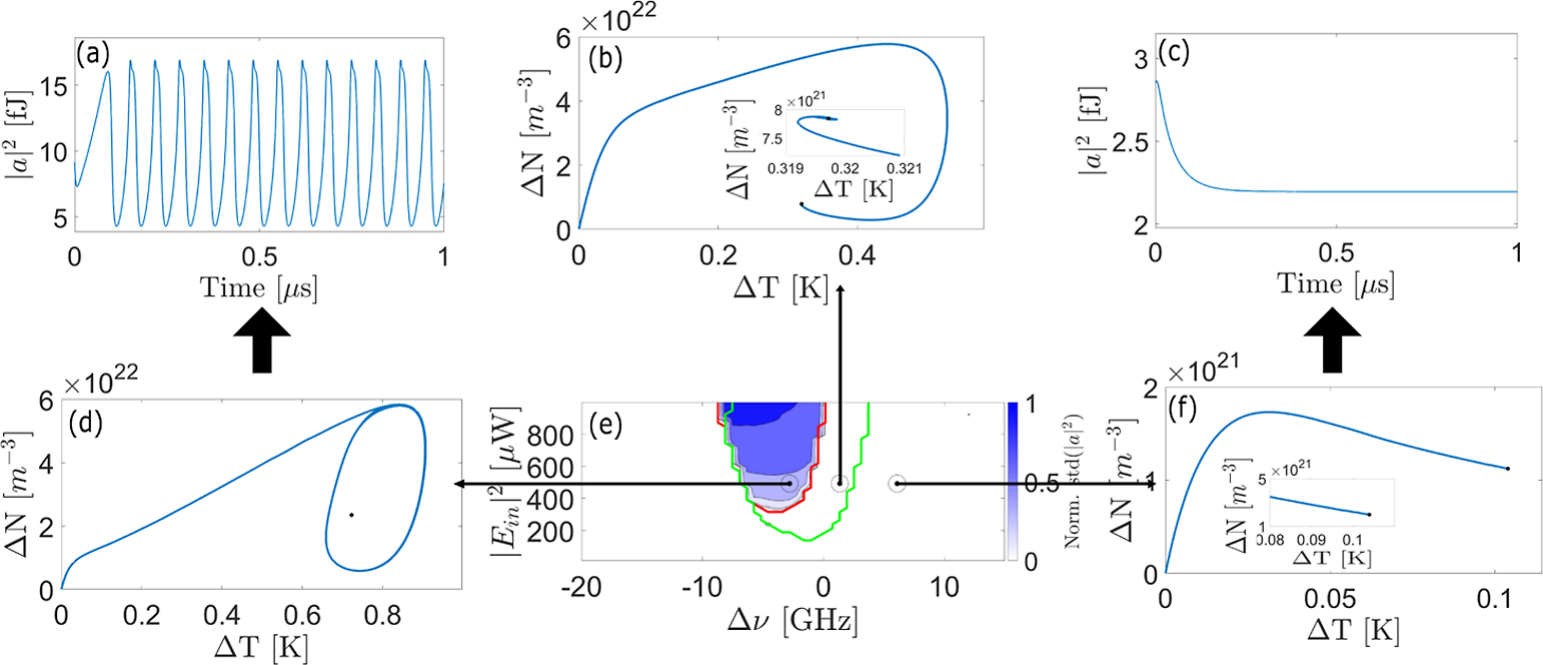
(a,c) MRR internal energy as a function of time for the control parameters (*P*_in_ = |*E*_in_|^2^ input power and Δν detuning with respect to the cold microring resonance) fixed by the left/right black markers in (e). (b,d,f) Phase portraits of the system for the control parameters fixed by the left/middle/right marker in (e), respectively. The phase portraits (blue lines) show the temporal evolution (trajectories) of Δ*N* (excess free carrier density within the MRR) and Δ*T* (temperature variation within the MRR) after the input signal is switched on. A black circle in the various panels points to the equilibrium point for those control parameters. The various phase portraits are representative of a situation where at least one eigenvalue has a positive real part (d), a nonzero imaginary part and both negative real parts (b), and zero imaginary part, and both negative real parts (f). (d,a) Are representative of a self-pulsing regime (the corresponding phase portrait and oscillating internal energy are shown). A stable regime is obtained when the eigenvalues have a nonzero imaginary part and both negative real parts. This is represented by the (c,f) panels. (e) Color map of the standard deviation of the field intensity within the MRR normalized to 1 after the transient stage (6 μs) as a function of the control parameters. The system dynamics was computed with the Runge–Kutta algorithm. The input signal (*E*_in_) is a step function at *t* = 0. As a comparison, the green and red lines represent the results of the linearization method. These lines contour the control parameter space for which the imaginary part of the eigenvalues is different from zero and for which the real part of at least one eigenvalue is larger than zero, respectively.

Figure 3e illustrates the standard deviation of the MRR energy after the transient phase (beyond the first 6 μs, as seen in Figure 3a–c). In regions of self-pulsing, a high standard deviation is observed, highlighting the range of control parameters where Runge–Kutta simulations indicate self-pulsing behavior. The colored region in Figure 3e corresponds to this self-pulsing regime. Notably, the eigenvalue analysis successfully predicts the self-pulsing region, as indicated by the red contour that encompasses most of the region where the standard deviation differs from zero. Minor discrepancies may arise due to slow decay dynamics or numerical approximations in determining the equilibrium values used to calculate eigenvalues. When the control parameters produce a stable equilibrium, the nature of the system’s decay is determined by the presence or absence of an imaginary component in the eigenvalues. If the eigenvalues have an imaginary part, the decay dynamics (Figure 3b) exhibit damped oscillations. Conversely, if there is no imaginary part, the decay is nonoscillatory (Figure 3f), and the system settles into equilibrium as predicted by eq 4. If the control parameters lead to an unstable equilibrium, the energy dynamics within the cavity become unstable. In this case, the system exhibits periodic oscillations, represented in the phase portrait as a stable orbit surrounding the unstable equilibrium point (Figure 3d). Correspondingly, the cavity energy oscillates as a function of time, as shown in Figure 3e. This is the case where self-pulsing in the through and drop-port transmission is observed. Furthermore, eq 4 reveals that the oscillation frequency of the MRR energy near an equilibrium point is related to $\frac{\left|Im\left(\lambda_{1}\right)\right|}{2\pi }$ . When self-pulsing is present, the system is not arbitrarily close to the equilibrium point where the linearization method holds. Hence, $\frac{\left|Im\left(\lambda_{1}\right)\right|}{2\pi }$ gives simply an estimate of the self-pulsing frequency which is the more accurate the nearer is the stable orbit to the equilibrium point.

### 2.3 Other Equilibrium Points

When additional equilibria are present (see e.g. Figure 1b green and red dots), the eigenvalue maps in Figure 2 can be evaluated at their corresponding values. The resulting maps are shown in Figure 4.

**Figure 4:**
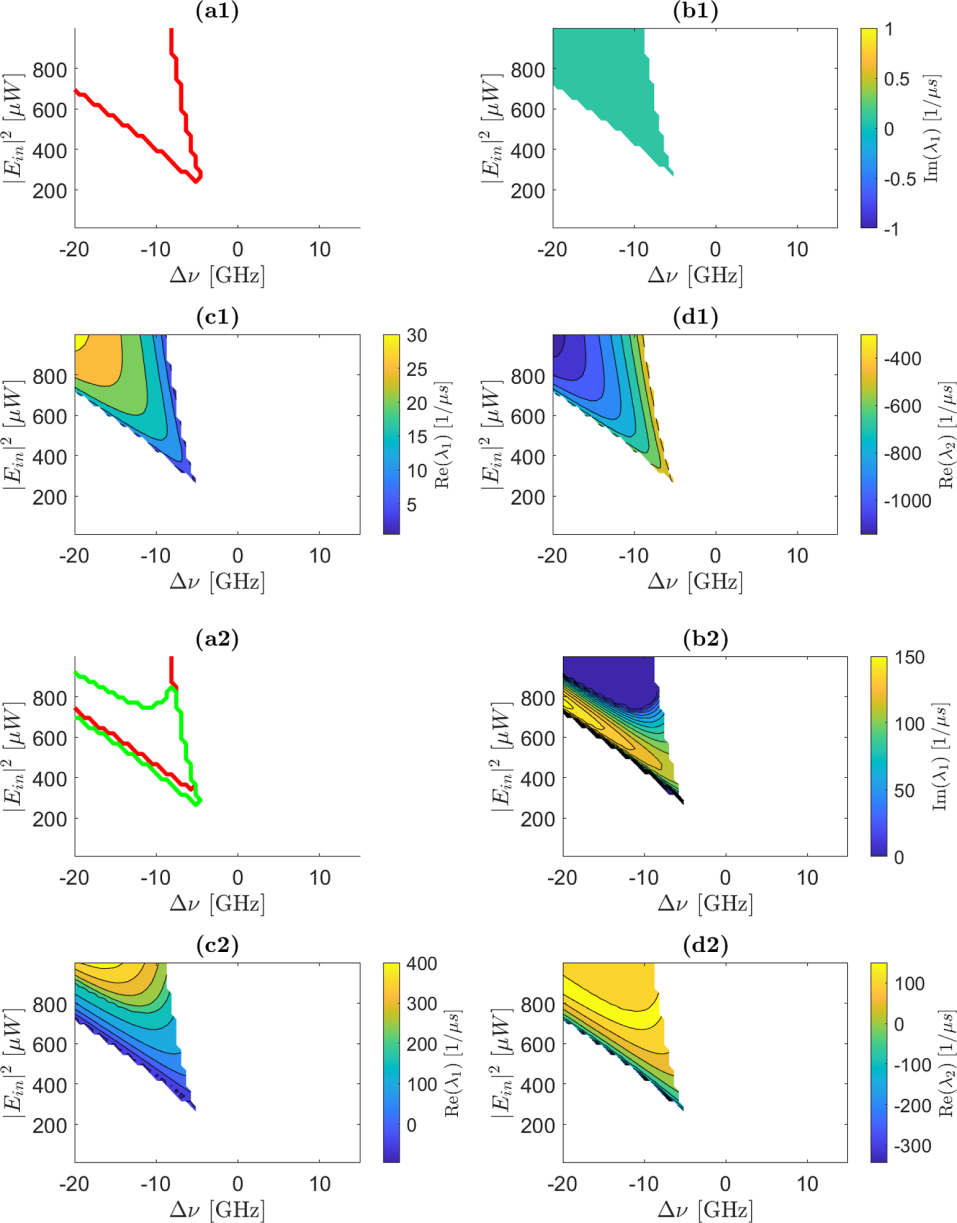
(a) Stability map of the microring as a function of the control parameters (*P*_in_ = |*E*_in_|^2^ input power and Δν detuning with respect to the cold microring resonance). The red and green lines divide the map in three regions. Within the region defined by the red line, at least one eigenvalue has a positive real part. Within the region outside the red and inside the green lines, the imaginary parts of the two eigenvalues are different from zero but the real parts are both negative. In the region exterior to the green and red line, the imaginary parts of the eigenvalues are equal to zero and the real parts negative. (b) Map of the imaginary part of the first eigenvalue as a function of the control parameters. (c,d) Maps of the real parts of the first and second eigenvalues, respectively. The color scales are given by the bars on the right of panels (b,c,d). Note that for the real parts, these span both positive and negative values. Each eigenvalue is calculated at the (a1–d1) second/(a2–d2) third equilibrium point. The white color in (b–d) means that only the first equilibrium point does exist for the corresponding control parameters.

Figure 4a1 shows that the second equilibrium is always unstable. In contrast, Figure 4a2 reveals that, while the third equilibrium is mostly unstable, a small region exists where it remains stable. Notably, there are several control parameter values for which the first equilibrium is stable while the third remains unstable.

In this scenario, the system’s dynamics depend on the initial conditions. If the system starts near the third equilibrium, it will become unstable. Conversely, if it starts near the first equilibrium, such as in cases with no input power, it will reach in a stable state. The system trajectories following these dynamics, computed using the Runge–Kutta algorithm, are shown in Figure 5left by the blue and yellow lines, respectively.

**Figure 5:**
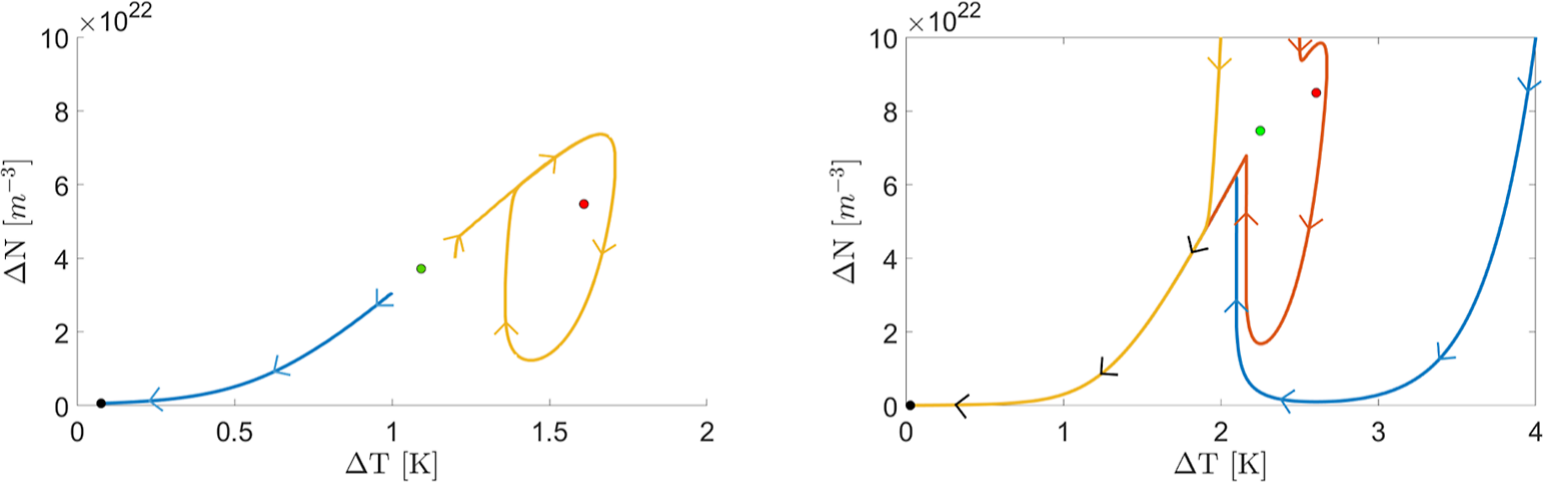
(left) Trajectory of the system in the (Δ*T*, Δ*N*) space (phase portraits) for an input power of 600 μW and a detuning from the cold resonance of −10 GHz. The two lines correspond to two different initial conditions: Δ*N* = 3 × 10^22^ m^–3^ and Δ*T* = 1 K for the blue line, and Δ*N* = 4 × 10^22^ m^–3^ and Δ*T* = 1.2 K for the yellow line. The black, green, and red dots mark the equilibrium points at the lowest, middle, and highest MRR internal energy, respectively. (right) Trajectories of the system in the (Δ*T*, Δ*N*) space for an input power of 800 μW and a detuning from the cold resonance of −19 GHz (a point in the control space which corresponds to the black marker in Figure 6). The initial conditions are Δ*N* = 1 × 10^23^ m^–3^ and different Δ*T* = 4 K (blue line), 2.5 K (red line), and 2 K (yellow line). (text color black) The black, green, and red dots mark the equilibrium points at the lowest, middle, and highest MRR internal energy, respectively.

We can evaluate whether the linearization and stability analysis at the third equilibrium reliably predicts the self-pulsing behavior observed using the Runge–Kutta algorithm when initialized near the third equilibrium point. This is done by evaluating a map similar to the one in Figure 3e, with the resulting map shown in Figure 6. It can be noticed that in some regions, as the one indicated by the black marker, the linearization method breaks down, i.e., its results do not align with the results of the full solution of eq ().We evaluated the evolution of Δ*T* and Δ*N* for different starting conditions at the black marker in Figure 6. The results are shown in Figure 5right. The trajectory in Figure 5right suggests that both the third and second equilibria are unstable. The initial condition lies in the attraction basin of the first equilibrium, and ultimately, the MRR state converges to it.

**Figure 6:**
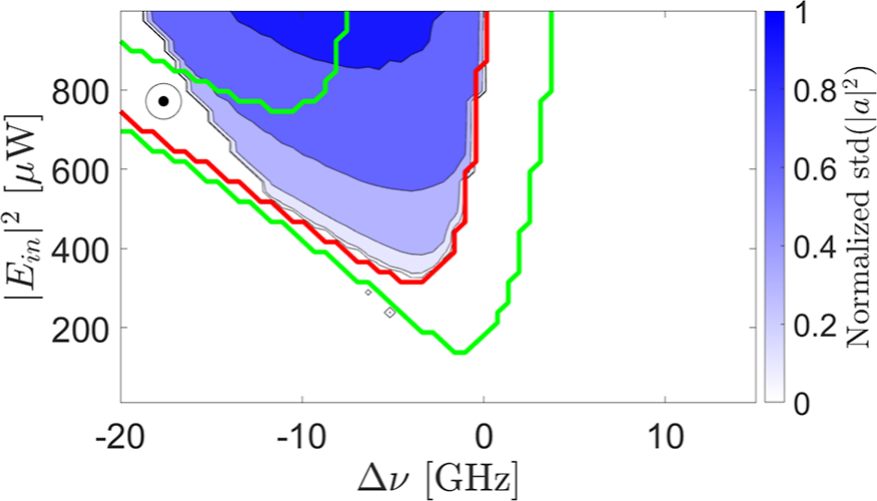
Color map of the normalized standard deviation of the energy (|*a*|^2^) in the MRR normalized after the transient stage (6 μs) as a function of the control parameters (*P*_in_ = |*E*_in_|^2^ input power and Δν detuning with respect to the cold microring resonance). The system dynamics was computed with the Runge–Kutta algorithm. The input signal (*E*_in_) is a step function at *t* = 0. The green and red lines contour the control parameter space, as computed by the linearization method, for which the imaginary part of the eigenvalues is different from zero and for which the real part of at least one eigenvalue is larger than zero, respectively. The black marker refers to Figure 5(right). The initial conditions of the Runge–Kutta algorithm are Δ*N* = 1 × 10^23^ m^–3^ and Δ*T* = 4 K.

Let us note that the dynamics associated with the second and third equilibria can be reached by using specific initial conditions. For example, a pump and probe approach, where a constant large input signal is overlapped by a weaker modulated signal, allows reaching the third equilibrium point and the associated dynamics. Examples of an application of these last initial conditions can be found in ref 19.

### 2.4 Nonlinear Microring as a Reservoir

We now turn to an application of the proposed method. MRRs are used as nodes in a time-multiplexed reservoir computing scheme (RC).^4–6^ This is shown schematically in Figure 7. Briefly, the input signal consists of a sequence of bits that are input into the nonlinear MRR. The transmitted signal is then undersampled at different times. The resulting samples are used as virtual nodes and are processed through ridge regression to map them to a prediction. The ridge regression is an algorithm that takes as input the output of the MRR and the target, together with a ridge parameter, and returns as output the prediction ? The weights in the ridge regression are learned by minimizing the difference between the prediction and the target defined by a task. We evaluated the performance of the MRR as a RC for various control parameter values, input bit modulation amplitudes, and durations. The nonlinear system described by eq 3 was solved using the Runge–Kutta algorithm, with the input signal being a pseudorandom binary sequence (PRBS) of order 6. The results are then compared with the predictions of the linearization and stability analysis of the system.

**Figure 7:**
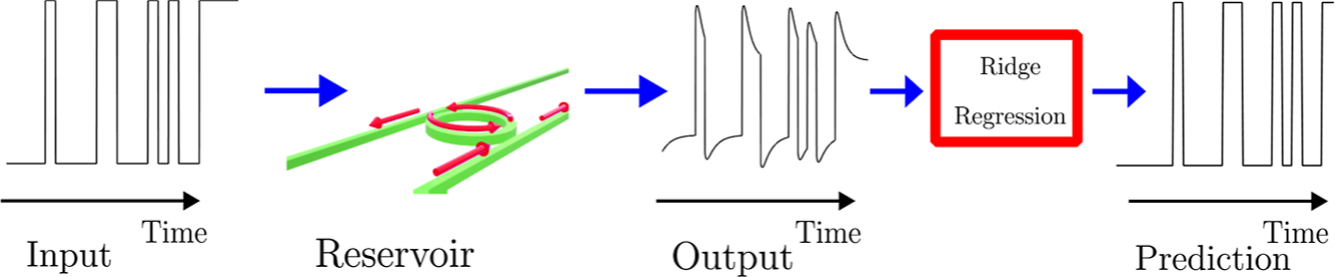
Scheme of a microring employed as a reservoir.

We applied the MRR as a reservoir to solve memory (MEM) tasks within an RC neural network. Let us call *x*(*n*) the bit input to the RC at time *n* and *y*(*n*) the output of the RC at time *n*. In the memory task, the RC has to output a bit at a time *n* equal to the one that was input at time *n* – 2, i.e., *y*(*n*) = *x*(*n* – 2). This task is named a delay-2 task. In^4^ we experimentally demonstrate the capability of a nonlinear MRR to solve memory and logical tasks, such as the AND or OR tasks, between the bit in the present (i.e., at time *n*) and a bit in the past (i.e., at time *n* – *j*, where *j* = 1, ..., 3). Here, we discuss only the MEM task because it allows capturing the role of the MRR nonlinear dynamics in the RC. In fact, in the delay-2 task, there are three bits to consider: the present bit (*x*(*n*)), the bit at time *n* – 1 (*x*(*n* – 1)) and the bit at time *n* – 2 (*x*(*n* – 2)). We can assume that the task is easier when *x*(*n*) = *x*(*n* – 2), namely if the present bit is equal to the bit that should be memorized by the RC. Indeed, in this case *y*(*n*) = *x*(*n*) = *x*(*n* – 2). Let us call a hard task for the RC when the present and the past bits are different. In this case the RC has to swap *y*(*n*) with respect to *x*(*n*) to perform the task, i.e. it has to force *y*(*n*) = *x*(*n* – 2). As a comparison to other logical tasks, the MEM task has more hard tasks. In fact, for the AND there are 8 possible combinations of the 3 relevant bits, where only two correspond to hard tasks (specifically the RC needs to flip a 1 to a 0). Similarly, for the OR task there are only two hard tasks (which require a flip from a 0 to a 1). On the contrary, for the MEM task we have four hard tasks which require to flip either a 1 to a 0 or a 0 to a 1. In addition, the delay-2 task has enough distance between the bits to make the task challenging enough to test our method. In fact, the delay-1 task would be too easy while the delay-3 task would require simply to tune the bit length to the MRR memory, which is an unnecessary complication with respect to the delay-2 task.

To test the performances of the RC, we also modulate the input according to a scheme (*x*, *y*), where the power associated with a binary 0 is *x* × |*E*_in_|^2^ and the power for a binary 1 is *y* × |*E*_in_|^2^ where |*E*_in_|^2^ represents the power shown on the *y*-axis of Figure 8. The Bit Error Rate (BER) is used as a figure of merit to quantify the difference between the prediction and the target.

**Figure 8:**
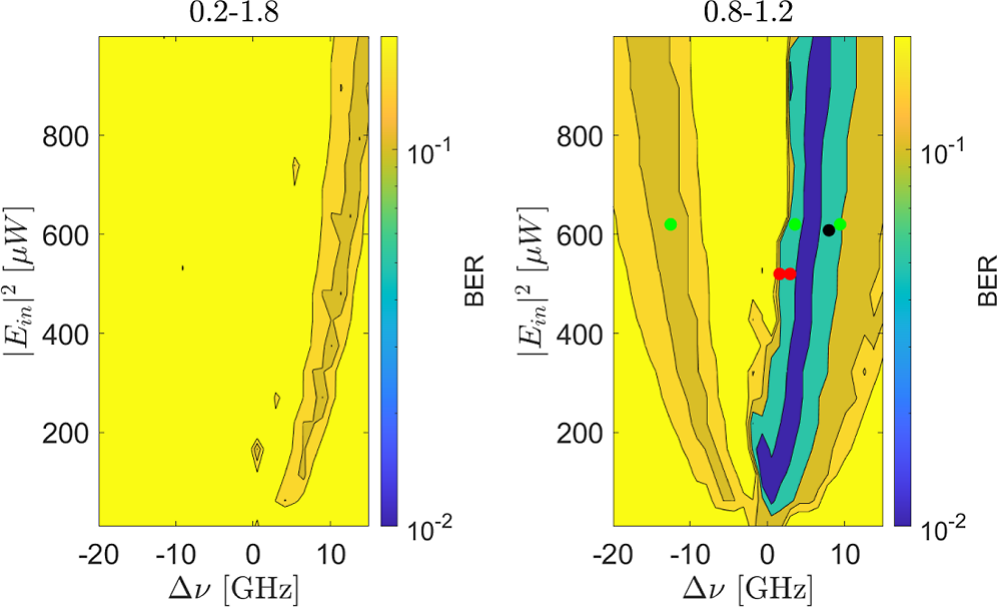
BER maps as a function of the control parameters for the MEM task. The input bit power level is modulated between (0.2–1.8) × |*E*_in_|^2^ (left) and (0.8–1.2) × |*E*_in_|^2^ (right). The input bit duration is 30 ns. The color markers in the right panel refer to Figure 9. Note that the BER scale is limited by the small data set we used to a low value of 0.015.

Figure 8 shows that small BERs are obtained with specific control parameter values. Surprisingly, the BER improves with low modulation amplitudes despite a worse signal-to-noise ratio (SNR). We interpret this trend with the dependence of the MRR dynamics on the control parameters. In fact, for small detunings, and at high input powers, self-pulsing impairs performance. For large detunings and when the input power is low, the MRR operates in the linear regime. In this regime, the free-carrier and thermo-optic times decouple and no longer depend on the control parameters. As a result, these times are not related to the bit length, negatively impacting performance. In contrast, at low input modulation amplitudes (right), the high power bit is characterized by a power for which the region where self-pulsing occurs is more restricted. Additionally, the free carrier and thermo-optic times remain coupled and depend on the control parameters. Consistently, the MRR dynamics align with the bit sequence and, thus, one finds low BERs.

Figure 9 provides valuable insights into the nonlinear response of the MRR to a modulated input signal within the stability analysis framework. It shows the phase portraits of Δ*N* and Δ*T* for a PRBS sequence. Figure 9a refers to a case when the dynamics of Δ*N* and Δ*T* within the MRR are coupled. Indeed, their evolution trajectory is neither purely vertical nor purely horizontal. Note that the various sequences of 1’s and 0 s in the PRBS input signal explain the complex shape observed. Moreover, we have marked in the figure with H or L the equilibrium points where the system will tend in case of a sequence of only 1’s (point H) or of only 0 s (point L). In contrast, Figure 9b illustrates a situation where their dynamics are decoupled, and the linear approximation does not hold for the entire phase space. When the input signal switches from a 1 to a 0 bit, the trajectory is initially almost vertical (indicating that the free carriers reach equilibrium first) and then almost horizontal (indicating that the temperature equilibrates independently, without affecting the free carrier concentration). The fact that we exit the linear approximation makes the upper-right angle of the trajectory in Figure 9b larger. Figure 9c compares two trajectories for control parameters which yield two different kinds of eigenvalues: the blue curve is characteristic for control parameters which yield an eigenvalue with a zero imaginary part no oscillation as described in Figure 3f while the orange curve for control parameters which yield eigenvalues with a nonzero imaginary part (as in Figure 3c). As already discussed in Section 2.2, the MRR dynamics with a nonzero imaginary part eigenvalue show a dumped oscillatory behavior as evidenced by the kink in the phase portrait which tends to the H equilibrium point.

**Figure 9:**
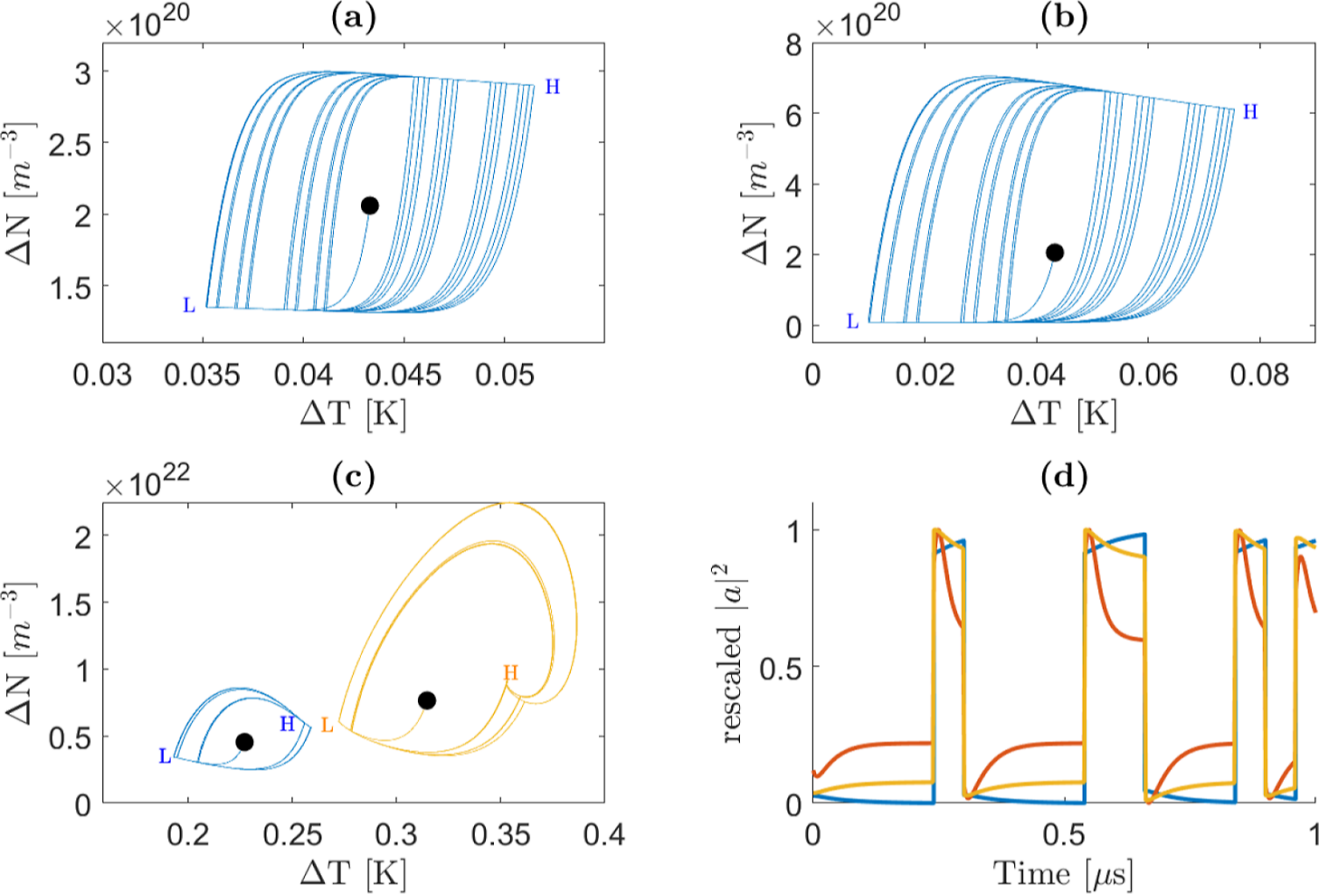
Phase portraits showing the trajectories of Δ*N* and Δ*T* for different pseudorandom binary sequences (PRBS) of order 6. Black dots set the initial conditions. Letters H and L refers to the high and low equilibrium points, to where the system evolves in case of an input sequence of only 1′s or 0s, respectively. Panel (a) the input signal is modulated between (0.8–1.2) × 610 μW and Δν = 7.96 GHz. Panel (b) input signal modulation (0.2–1.8) × 610 μW and Δν = 7.96 GHz (black marker in Figure 8). Panel (c) input modulation of (0.8–1.2) × 520 μW and Δν = 2.94 GHz (blue line) or Δν = 1.57 GHz (orange line). These two conditions are indicated by red markers in Figure 8. Panel (d) shows the temporal dependence of the microring stored energy (|*a*|^2^, rescaled between the minimum to zero and the maximum to 1, where *a* is the microring optical mode) for an input signal modulation of (0.8–1.2) × 620 μW and Δν = −12.53 GHz (blue line), 3.56 GHz (red line), and 9.41 GHz (orange line). These input control parameters are labeled with green markers in Figure 8. All bit lengths are 60 ns.

Finally, Figure 9d depicts the |*a*|^2^ dynamics for different Δν: negative (blue line), small positive (red line), and large positive (orange line). In all these cases, the control parameters yield a zero imaginary part of the eigenvalues, i.e., no oscillatory behavior. For a negative Δν, during the low-to-high transition, the increase in the input power raises the energy inside the MRR. This, in turn, causes an increase in both the free carrier concentration and the temperature. In this case, the two remain correlated and the temperature’s stronger influence dominates the overall warm resonance position of the MRR. As a result, we have a redshift of the MRR resonance, which locks the resonance frequency to the input frequency. For a positive Δν, the Δ*N* and Δ*T* dynamics are more complex. During the low-to-high transition, initially, the free carrier concentration rises, causing a blue shift of the MRR resonance. However, free carrier recombination causes a rise in the temperature, which in turn causes a redshift of the resonance. Therefore, the MRR internal field decreases. As the energy decreases the free-carrier concentration decreases. Despite this, the temperature continues to increase due to thermal inertia until it finally stabilizes. Additionally, the relaxation times for small positive Δν are shorter than for large positive Δν (compare yellow and orange lines in Figure 9d), which is consistent with the eigenvalue maps shown in Figure 2.

Figure 10 describes the effect of the bit length on the performance of the RC to solve the MEM task. It shows that the MEM task can be effectively solved when the input bit length falls within the range of 20 ns–70 ns. However, the optimal performance region depends on the specific bit length. The figure suggests that the oscillatory dynamics impedes solving the task, as evidenced by the high BER within the green contour. Additionally, for short bit lengths (20 ns–30 ns) better performances are achieved for small positive Δν. Conversely, longer bit lengths (60 ns–70 ns) perform better with large Δν. These behaviors are coherent with the map of the real part of the first eigenvalue obtained by the stability analysis. Indeed, the real part of the eigenvalue can be interpreted as the inverse of the system’s typical decay time. The real part eigenvalue map reveals low negative values in the region where the RC solves the MEM task for short bit lengths. The observed correlation between task performance and eigenvalue values implies that one can infer valuable information about the ideal bit length for a given pair of control parameters by simply analyzing the maps of the real and imaginary parts of the eigenvalues.

**Figure 10:**
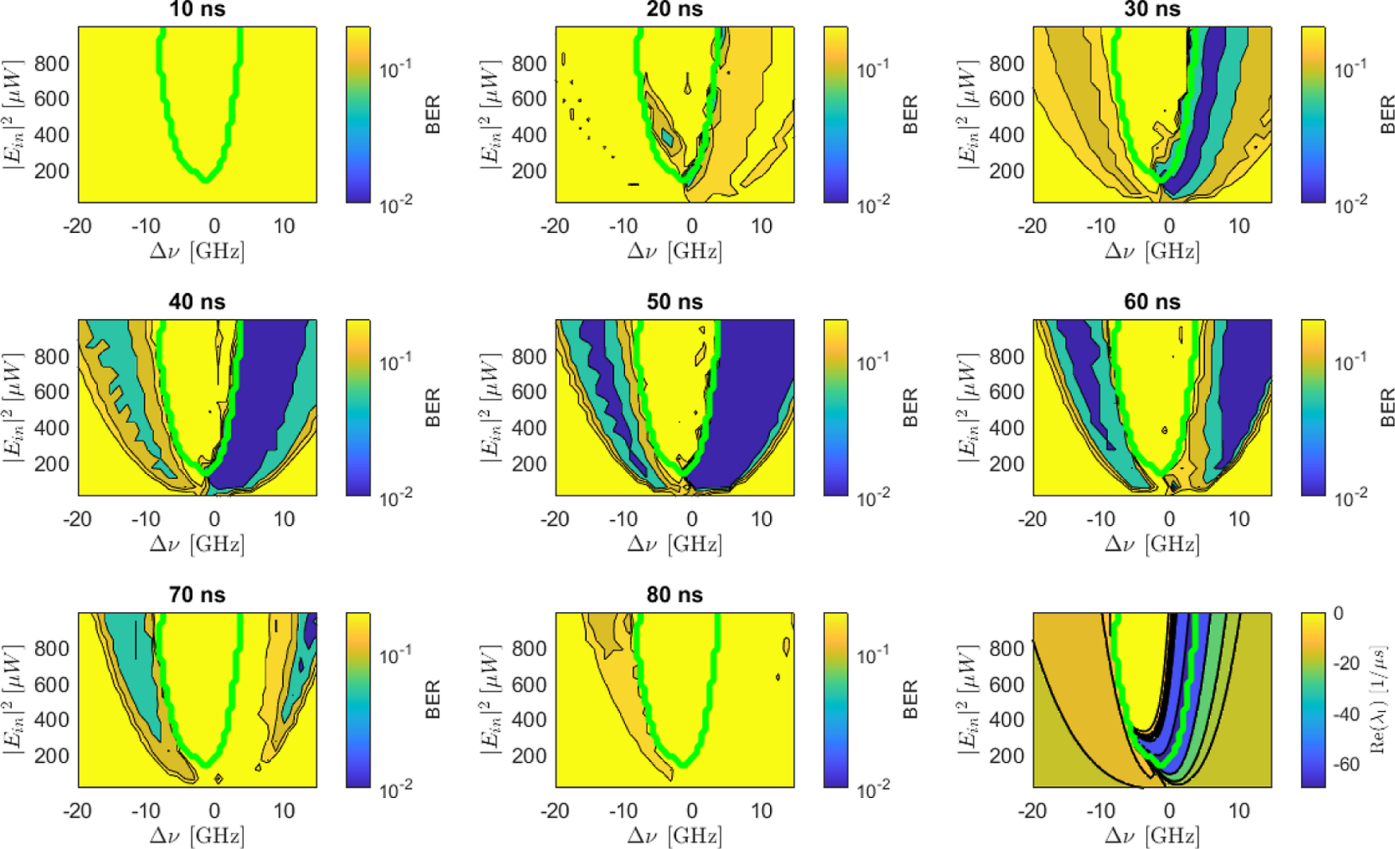
BER maps as a function of the control parameters for a MEM task with delay 2. The input signal is modulated between (0.8–1.2) × |*E*_in_|^2^. The different panels refer to different durations of the bits whose value is reported on each panel. The microring response is simulated by numerically solving the nonlinear eq 3. The bottom-right panel shows a color map of the real part of the first eigenvalue as a function of the control parameters. The green contour encloses the area for which the imaginary part of the eigenvalue is different from zero.

## 3 Conclusion

In conclusion, we have demonstrated that the eigenvalues of the Jacobian obtained by linearizing the nonlinear equations that describe the temporal evolution of the optical field of a MRR near equilibrium points are closely related to its internal dynamics. The knowledge of the eigenvalues allows the identification of the three distinct regions within the control parameter space that are characterized by a specific dynamical behavior. This result complements previous modeling of MRR where a bifurcation analysis of the excitability of MRR has been performed.^16^ Additionally, we confirmed that certain dynamical regimes of MRR prove more effective for task-solving applications in RC applications. These regimes can be anticipated through the eigenvalue analysis, offering a significant reduction in computational effort compared to directly solving the system dynamics using the Runge–Kutta algorithm. Furthermore, we demonstrated that maintaining similar powers at the lower and upper states of the input signal improves performance in solving the task.

The method presented here can potentially be extended to a sequence of multiple MRRs, such as the SCISSOR (side coupled integrated space sequence of resonators) or the CROW (coupled resonator optical waveguide) architectures. These systems involve significantly more degrees of freedom, making numerical simulations computationally expensive and time-intensive. The same challenges apply to the linearization and stability analysis method with the additional complication of identifying the system equilibrium points. As an example, to treat a two-ring CROW system, one needs to include additional equations for the second ring’s free-carrier concentration and temperature, as well as interaction terms accounting for power transfer between the rings. Preliminary efforts in this direction indicate that detecting multiple equilibrium points is extremely difficult due to the increasing complexity of the mathematical equations. If we consider n rings, we can expect that the time to solve the dynamics of the system by the Runge–Kutta method linearly increases with n. However, as mathematical complexity grows, our method may become impractical even for just three rings. Extending the linearization and stability analysis method to accommodate more rings is a critical challenge that demands dedicated theoretical and analytical effort—one that goes beyond the scope of this work. Whether such an approach will ultimately prove successful remains uncertain.

## Figures and Tables

**Figure 11:**
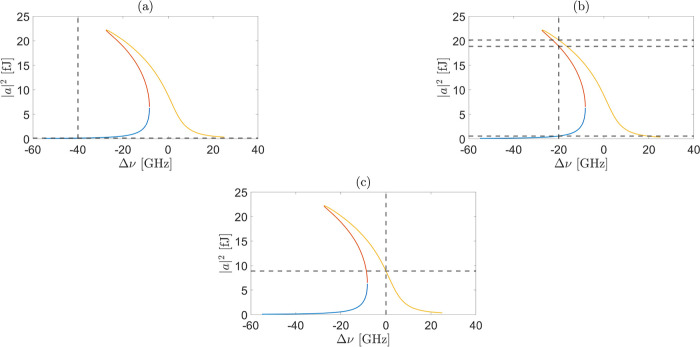
*E*_in_ isocurves in the ${\left|a\right|}^{2}$ and Δν plan. A different color corresponds to a different curve which solves the equation ${\left|E_{in}\right|}^{2}=900\;\mu W$ as a function of Δν. The dashed lines refer to (a) Δν = −40 GHz, (b) Δν = −20 GHz, and (c) Δν = 0 GHz.

## Appendix A: Methods

### A.1. A solution for the cavity energy

After setting the time derivative of the MRR optical field to zero in eqs ([Disp-formula eq1a]–[Disp-formula eq1c]c), it is possible to insert γ_loss_ from eqs ([Disp-formula eq2a]–[Disp-formula eq2c]c) and write

5
$$\begin{array}{l} \frac{da}{dt}=0=\left(i\left(\omega_{r}+\delta \omega_{nl}-\omega \right)-\frac{2\gamma_{coup}+\gamma_{rad}+\gamma_{abs,lin}+\frac{\Gamma_{TPA}\beta_{Si}c^{2}}{n_{g}^{2}V_{TPA}}|a{|}^{2}+\frac{\Gamma_{FCA}\sigma_{Si}c}{n_{g}}\Delta N}{2}\right)a+i\sqrt{\gamma_{coup}}E_{in}\\ \Rightarrow \gamma_{coup}{\left|E_{in}\right|}^{2}=\left[{\left(\omega_{r}+\delta \omega_{nl}-\omega \right)}^{2}+{\left(\frac{2\gamma_{coup}+\gamma_{rad}+\gamma_{abs,lin}+\frac{\Gamma_{TPA}\beta_{Si}c^{2}}{n_{g}^{2}V_{TPA}}|a{|}^{2}+\frac{\Gamma_{FCA}\sigma_{Si}c}{n_{g}}\Delta N}{2}\right)}^{2}\right]{\left|a\right|}^{2} \end{array}$$

It is now easy to see that eq 5 is a cubic of |*a*|^2^. In fact, it can be rewritten as

6
$$c_{0}\left(\Delta T,\Delta N\right)+c_{1}\left(\Delta T,\Delta N\right)\left|a{|}^{2}+c_{2}\left(\Delta T,\Delta N\right)\right|a{|}^{4}+c_{3}\left(\Delta T,\Delta N\right)|a{|}^{6}=0$$

After some algebraic steps, it is possible to find an explicit solution for |*a*|^2^ as a function of (Δ*T*, Δ*N*). We used Wolfram Mathematica to find the solutions. The first reads

7
$${\left|a\right|}^{2}=-\frac{\sqrt[{3}]{2}\left(3c_{1}c_{3}-c_{2}^{2}\right)}{3c_{3}\sqrt[{3}]{\sqrt{\Delta }+\phi }}+\frac{\sqrt[{3}]{\sqrt{\Delta }+\phi }}{3\sqrt[{3}]{2}c_{3}}-\frac{c_{2}}{3c_{3}}$$

where

8
$$\Delta \equiv {\left(9c_{1}c_{2}c_{3}-27c_{3}^{2}c_{0}-2c_{2}^{3}\right)}^{2}+4{\left(3c_{1}c_{3}-c_{2}^{2}\right)}^{3},,\phi \equiv 9c_{1}c_{2}c_{3}-27c_{3}^{2}c_{0}-2c_{2}^{3}$$

The other two solutions are complex for the parameters we consider, meaning we can discard them as nonphysical.

### A.2 Detection of equilibria

The equilibria are values of (|*a*|^2^, Δ*N*, Δ*T*) for which all the time derivatives in eqs ([Disp-formula eq1a]–[Disp-formula eq1c]c) are equal to zero. When trying to evaluate the equilibria analytically, one finds a *n* > 4 degree polynomial, for which a general solution is not available. A numerical approach is required.Here, we used the following method. We set the time derivatives of Δ*N* and Δ*T* to zero and evaluate them as functions of |*a*|^2^

9
$$\begin{array}{ll} \Delta N_{eq}\left(\Delta \nu ,|E_{in}{|}^{2}\right) & =\tau_{fc}\frac{\Gamma_{FCA}\beta_{Si}c^{2}}{2\hbar \omega V_{FCA}^{2}n_{g}^{2}}{\left|a\right|}^{4}\\ \Delta T_{eq}\left(\Delta \nu ,|E_{in}{|}^{2}\right) & =\tau_{th}\frac{\Gamma_{th}\gamma_{abs}\left(|a{|}^{2},\Delta N_{eq}\right)}{\rho_{Si}c_{p,Si}V_{th}}{\left|a\right|}^{2} \end{array}$$

Then, we insert these results into eq 5 and evaluate |*E*_in_|^2^ for a 2D grid of |*a*|^2^ and Δν values. Now, we interpolate a curve of |*a*|^2^ vs Δν for a chosen value of input power to get curves like those in Figure 11.

We can see that each intersection with the vertical line in Figure 11 indicates the presence of an equilibrium point of |*a*|^2^ for a given pair of control parameters, i.e. *E*_in_ and Δν. For the control parameters considered in Figure 11b, the system has three equilibrium points. In the other plots, only one equilibrium point is present. Figure 11 shows that the system always admits at least one equilibrium point and at most three.

After choosing *E*_in_ and Δν, we obtain the equilibrium value of ${\left|a\right|}^{2}$ for the given pair of control parameters by means of an interpolation. Once we got ${\left|a_{eq}\left(\Delta \nu ,{\left|E_{in}\right|}^{2}\right)\right|}^{2}$ , we can insert it into eq 9 to get the equilibrium values Δ*N*_eq_ and Δ*T*_eq_.

### A.3 Eigenvalues detection

To evaluate the eigenvalues, one has to evaluate the elements of the Jacobian of the linearized system, namely from eq 3

10
$$\begin{array}{l} J_{11}=\frac{\partial F_{N}}{\partial \Delta N}=-\frac{1}{\tau_{fc}}+2\Gamma_{FCA}\beta_{Si}\frac{c^{2}}{2\hbar \omega_{r}V_{FCA}^{2}n_{g}^{2}}{\left|a\right|}^{2}\frac{\partial |a{|}^{2}}{\partial N}\\ J_{12}=\frac{\partial F_{N}}{\partial \Delta T}=2\Gamma_{FCA}\beta_{Si}\frac{c^{2}}{2\hbar \omega_{r}V_{FCA}^{2}n_{g}^{2}}{\left|a\right|}^{2}\frac{\partial |a{|}^{2}}{\partial T}\\ J_{21}=\frac{\partial F_{T}}{\partial \Delta N}=\frac{\partial T_{a2}}{\partial N}{\left|a\right|}^{2}+T_{a2}\frac{\partial |a{|}^{2}}{\partial N}\\ J_{22}=\frac{\partial F_{T}}{\partial \Delta T}=-\frac{1}{\tau_{th}}+\frac{\partial T_{a2}}{\partial T}{\left|a\right|}^{2}+T_{a2}\frac{\partial |a{|}^{2}}{\partial T} \end{array}$$

where

11
$$T_{a2}=\Gamma_{th}\frac{\gamma_{abs}}{\rho_{Si}c_{\rho ,Si}V_{th}}$$

In this way, the Jacobian may be expressed as

$$\mathrm{J⃡}=\left(\begin{array}{ll} J_{11} & J_{12}\\ J_{21} & J_{22} \end{array}\right)$$

and the eigenvalues can be easily obtained by solving

12
$$\begin{array}{l} \det\left[(\begin{array}{ll} J_{11}-\lambda & J_{12}\\ J_{21} & J_{22}-\lambda \end{array})\right]=0\\ \Rightarrow \lambda_{1,2}=\frac{1}{2}\left(J_{11}+J_{22}\pm \sqrt{-2J_{11}J_{22}+J_{11}^{2}+4J_{12}J_{21}+J_{22}^{2}}\right) \end{array}$$

Since the elements of the Jacobian are real, one can see that the eigenvalues are complex only if the argument of the squared root in eq 12 is smaller than zero. In this case, the imaginary part of the two eigenvalues is equal to

$$Im\left(\lambda_{1,2}\right)=\pm \frac{1}{2}\sqrt{2J_{11}J_{22}-J_{11}^{2}-4J_{12}J_{21}-J_{22}^{2}}$$

### A.4 Constants

Table 1 lists the meanings and values of the numerical constants in eq (). The values used were taken from ref 16.

**Table 1:** Parameters Employed for the Simulations

Planck constant over 2π	*ℏ*	1.05 × 10^–43^ g m^2^/ps
speed of light in vacuum	*c*	299,792,458 × 10^–12^ m/ps
resonant frequency	ω_r_	2π × 193.069 THz
TPA constant	β_Si_	8.4 × 10^–12^ mW^–1^
thermo optic coefficient of silicon	$\frac{dn_{Si}}{dT}$	1.86 × 10^–4^ K^–1^
lumped free carrier dispersion coefficient	$\frac{dn_{Si}}{dN}$	–1.73 × 10^–27^ m^3^
absorption cross-section	σ_Si_	10^–21^ m^2^
silicon density	ρ_Si_	2.33 g cm^–3^
silicon thermal capacity	*c*_p,Si_	0.7 J g^–1^ K^–1^
refractive index of bulk silicon	*n*_g_ = *n*_Si_	3.476
efficiency of linear absorption rate	$\eta_{lin}=\frac{\gamma_{abs,lin}}{\gamma_{abs,lin}+\gamma_{rad}}$	0.4
linear absorption rate	γ_abs,lin_	$\frac{\eta_{lin}}{102.5}THz$
coupling loss into the waveguide	γ_coup_	0.0098 THz
thermal relaxation time	τ_th_	65 ns
free carriers relaxation time	τ_fc_	5.3 ns
thermal confinement factor	Γ_th_	0.9355
TPA confinement factor	Γ_TPA_	0.9964
FCA confinement factor	Γ_FCA_	0.9996
Thermal effective volume	*V*_th_	3.19 μm^3^
TPA effective volume	*V*_TPA_	2.59 μm^3^
FCA effective volume	*V*_FCA_	2.36 μm^3^

## †

LIGENTEC SA, Chemin de la Dent-d’Oche 1B 1024 Ecublens VD, Switzerland
